# Intellectual and Developmental Disabilities: Eugenics

**DOI:** 10.3389/fpubh.2014.00201

**Published:** 2014-10-20

**Authors:** Ingrid Grenon, Joav Merrick

**Affiliations:** ^1^Formerly affiliated with Wrentham Developmental Center, Massachusetts Department of Developmental Services, Wrentham, MA, USA; ^2^Division for Intellectual and Developmental Disabilities, Ministry of Social Affairs and Social Services, Health Services, Jerusalem, Israel

**Keywords:** intellectual disability, eugenics, developmental disabilities, history, social darwinism

## Introduction

Those working today with individuals with intellectual and developmental disabilities (IDD) are often not aware of the dark side of our history concerning the treatment of this population and why we, some 150 years ago, made institutions or state schools for them (1).

Maybe we have forgotten or chosen to forget something that we were not taught in school, and it is likely that our teachers did not know it either.

## Eugenics

In 1883, Sir Francis Galton (1822–1911), cousin of Charles Darwin (1809–1882), coined the term “eugenics.” In his book “Inquiries into human faculty and its development” from 1883, Galton loosely defines eugenics as “the cultivation of race,” or “the science of improving stock” (2).

Eugenics is among many late nineteenth century ideologies encompassed in the term Social Darwinism. This coincided, interestingly, with the Progressive Era, which occurred in the period roughly from the 1890s into the 1920s in the United States. So-called “Progressives” were responsible for the Food and Drug Act in 1906, Prohibition in 1919, and women’s right to vote in 1920.

Galton’s new science spread like a wildfire in the United Kingdom and the United States and in 1907 Indiana passed the first law allowing “undesirables and defectives,” such as the “mentally retarded,” to be involuntarily sterilized. This is considered to be the first such eugenic “law” to be passed in the world. By 1909, California had passed laws permitting the sterilization of “undesirables.” Terms, such as mental hygiene, racial hygiene, social hygiene, and racial and human betterment became prevalent. Unbelievably, the US was a hotbed of racial purists striving to protect their master race at the time.

All of this coincided nicely with French psychologist Alfred Binet (1857–1911) and his test to measure intelligence, first published in 1905. The Binet test, originally intended to identify mentally retarded children within the school system, strengthened the eugenics movement by giving it additional impetus.

In 1906, Dr. Henry Herbert Goddard (1866–1957) was the Director of the Research Department at the Training School for the feeble-minded in Vineland, NJ, USA. Opening its doors in March of 1888, the Training School in Vineland was considered to be the third institution of its kind; the first opened in Massachusetts in 1848 and the second in New York in 1852. It was at the New Jersey Training School that Binet’s Intelligence Test was translated from French to English and readied for practical application under Goddard’s direction. Interestingly, it was also Dr. Goddard who coined the term “moron.” (Goddard derived “moron” from the ancient Greek “moros,” meaning dull, foolish, or sinful). Binet’s test became known as an IQ test.

In 1912, “The Kallikak family” (3) written by Dr. Goddard was published, and soon became a bible of sorts for proponents of eugenics (4). This book, and many others like it, became best sellers of the time. The “Kallikak family” is significant because it was the first of its kind, and certainly quite popular. In his book, Goddard warns of a “rising tide of feeble-mindedness,” and urges his readers to take action lest a “hereditary taint” should become the ruination of our society.

This work portrays, according to Goddard, an actual case study of the family of one of the feeble-minded residents under his care at the Training School. He gives her a fictional name, Deborah Kallikak, and goes on to trace her family back to her great–great–great grandfather Martin Kallikak Sr., a Revolutionary War soldier who had an unfortunate liaison with a young woman who was “feeble-minded and degenerate.” This faux pas, according to Goddard, led to “an appalling amount of defectives.” The descendants numbered 480, and of those at least 332 were determined to be “defective” according to Goddard’s research methodology.

The results of Goddard’s study, according to Goddard, proved that feeble-mindedness was passed from one generation to the next. He warned the reader in his final chapter, in regard to the woman with whom Martin Kallikak Sr. had been indiscreet, “When we conclude that had the nameless girl been segregated in an institution, this defective family would not have existed.” Regarding just the Kallikak family, Goddard further adds, “Society had to pay the heavy price.” The good doctor again cautions the reader by reminding them, “There are Kallikak families all about us – they are multiplying at twice the rate of the general population.” Goddard fanned the flames of the eugenic fire until it raged out of control. It should be no surprise, with this book on the best seller list and taken very seriously indeed, there was a surge in the sterilization frenzy. Massachusetts, however, was one of the few states that did not have mandatory sterilization laws.

A myriad of books with the same theme seem to have emerged between 1912 and 1930, all touting the necessity of eugenics and giving ample warning of how our society would degenerate if its principles were not followed. “Safe counsel or practical eugenics” (5) published in 1922, not surprisingly in Illinois, was a manual encouraging young men and women to find “suitable” partners in marriage in order to “improve the human race.” In addition, the book advocates for “sterilization of the feeble-minded, degenerate, and criminal.” A study of the feeble-minded (6) published in 1920, addressed the “national problem of the mental defective” and specifically stated that the “mentally defective” are those who are not able to have a home and for whom “the only permanent parent is the State.”

From California, the first hotbed of eugenics proponents, Ezra S. Gosney (1855–1942) and Paul Popenoe (1888–1979) brought us “Sterilization for human betterment” (7), a book chronicling 6,000 “successful” sterilizations in California of “idiots and other undesirables.” According to Gosney “eugenic sterilization, primarily, is applied by the state or with its sanction, to persons who would be likely to produce defective children.”

In 1911, when Dr. Goddard was busy studying the bloodlines of some of the residents at the Training School in New Jersey, the Governor of that state, Woodrow Wilson (1856–1924) and later 28th US President, signed a sterilization bill that went into law. The new 1911 law specifically stated, “An act to authorize and provide for the sterilization of feeble-minded, epileptics, rapists, certain criminals, and other defectives.”

Keeping in step with the rest of the nation, eugenics was all the rage in Massachusetts. Often touted as a progressive state, during this period Massachusetts was in many respects a bastion of backward thinking, at least by today’s standards. We found a small pamphlet from the “Massachusetts Society for Prevention of Cruelty to Children” on “The menace of the feeble-minded in Massachusetts” (8).

A growing public sentiment requires that feeble-minded women of child-bearing age who are a burden to the community and a menace to the future well being of the race, and defectives with criminal tendencies shall be segregated … Feeble-mindedness is 80% hereditary. By segregation only can sufficient relief be obtained so that these unfortunates will not propagate their kind … Segregation is humane and effective, and a good financial investment for developing greater prosperity and happiness in the future.

In January of 1922, Massachusetts Governor Channing Cox (1879–1968) addressed the Massachusetts Legislature and the following is a bit of what he said:

The Commonwealth has recognized the importance of a practical mental hygiene program, and had provided much legislation to make effective such a program. The State’s program for the feeble-minded embraces the following factors:

- Identification
- Registration
- Education
- Supervision
- Segregation

Of course, segregation was important in this age of eugenics, and interestingly the third Massachusetts “state school,” Belchertown, would have opened the same year. Just 1 year earlier, in September of 1921, Cox made history when he became the first Massachusetts governor to broadcast live. His radio debut was made in Springfield, MA, USA at the Eastern States Exhibition. Interestingly, during the 1920s “Fitter family” contests sponsored by the American Eugenics Society were quite popular at community fairs and livestock expositions. This was especially so in Massachusetts at the Eastern States Exhibition, where the best-bred humans were shown off alongside their counterparts from the animal kingdom.

The United States was certainly setting the trend in eugenics, and in so doing gaining the world’s attention. By the end of the second decade of the twentieth century, eugenics was so well-rooted in this country that it seems to have become the *status quo*. In 1927, Supreme Court Justice Oliver Wendell Holmes (1841–1935), a staunch supporter of eugenics, ruled in favor of the compulsory sterilization of a young woman in Virginia. The case, known as Buck v. Bell, served to authorize the legitimacy of the sterilization of defectives within the United States, especially those who were institutionalized (4). To summarize the litigation; in 1924, the superintendent of a Virginia state school, Dr. John Hendren Bell, requested permission from the school’s Board of Directors to have 18-year-old Carrie Buck sterilized, because in his opinion she was both feeble-minded and promiscuous. It would seem that Miss Buck had given birth to an illegitimate child, and that both her mother and grandmother had been suspected of being mentally retarded. What is really notable is what the Supreme Court Justice had to say about the case:

It is better for all the world, if instead of waiting to execute degenerate offspring for crime or to let them starve for their imbecility, society can prevent those who are manifestly unfit from continuing their kind…three generations of imbeciles are enough.

## Conclusion

It is sufficient to say that eugenics was not only fashionable; it had somehow become the law of the land. There were so many eugenics devotees lurking about during the early part of the last century that it would require a whole volume just to name them all.

The eugenics movement began to spread from the United States to Germany and there was one person who recognized its dark potential, and began to orchestrate a plan to put it to full use (4). His name was Adolph Hitler. It is indeed frightening to think that Hitler got his visions of racial purity from ideas popularized both in the United Kingdom and the United States of America, but he did. A proponent of American eugenics, Adolph Hitler adopted this strategy and began to put it to work in his native country. The American best seller “Sterilization for human betterment” was reprinted in Germany in 1933. In Germany, the first involuntary sterilization laws were put into effect in 1934, 27 years after the first US laws were passed (9).

Although some may find this topic disturbing and would prefer a more pleasant subject, it seems that it is a matter that must be exposed over and over, lest we forget.

Those who cannot remember the past are condemned to repeat it George Santayana (1863–1952), Spanish philosopher and novelist

## Conflict of Interest Statement

The authors declare that the research was conducted in the absence of any commercial or financial relationships that could be construed as a potential conflict of interest.
